# Supplementary material for: Precision and temporal dynamics in heading perception assessed by continuous psychophysics
Source: PLoS One. 2024 Oct 11;19(10):e0311992. doi: 10.1371/journal.pone.0311992 (PMC11469512; doi:10.1371/journal.pone.0311992)
Supplement: S1 Appendix — (DOCX) [file pone.0311992.s001.docx]

# Appendix – How Robust is This Method?

In order to get a better understanding of how robust this novel method is, we employed a three-pronged approach: first, we split our data set into three parts and performed the analyses above separately across all three subsets. This allowed us to assess the robustness of the method to between-participant variability (See A – Between Participant Variability) below. We also split each individual run from each participant into four parts of equal length to assess how performance varied across the course of one run (B – Within-Participant Variability: Over the Course of The Run). Finally, we performed a individual-level bootstrap analysis in which, for each participant, we randomly picked 25% of the frames on any given run and computed the Best Lag of Least Difference and the Least Difference for each of these subsets to assess within-participant variability (C – Within-Participant Variability: Robustness to Dropping Random Data Points).

.

### A – Between-Participant Variability

To assess between-participant variability, we split the dataset into three subsets of 10 participants each and performed our pre-registered analyses over these subsets. Table A1 contains the results of the bootstrap analysis, Table A2 displays the results of the LMM analysis, and Figure A1 shows the fitted noise parameters from the Kalman filter analysis.

Table A1: Results of bootstrap analysis

|  | *Difference Contrast* | *Confidence Interval (Lower Bound)* | *Confidence Interval (Upper Bound)* | *Significance* |
| --- | --- | --- | --- | --- |
|  | ***Time Lag of Least Difference*** | | | |
| FAST (vs. SLOW) |  |  |  |  |
| p131-p140 | **-0.25s** | **-0.5s** | **-0.08s** | ***** |
| p141-p150 | **-0.35s** | **-0.6s** | **-0.13s** | ***** |
| p151-p160 | **-0.16s** | **-0.55s** | **-0.03s** | ***** |
| DENSE (vs. SPARSE) |  |  |  |  |
| p131-p140 | **-0.31s** | **-0.5s** | **-0.13s** | ***** |
| p141-p150 | **-0.18s** | **-0.48s** | **0.07s** | **n.s.** |
| p151-p160 | **-0.09s** | **-0.34s** | **0.15s** | **n.s.** |
|  | ***Normalized Least Error (Variability in Performance)*** | | | |
| FAST (vs. SLOW) |  |  |  |  |
| p131-p140 | **-0.12** | **-0.15** | **-0.08** | ***** |
| p141-p150 | **-0.08** | **-0.14** | **-0.01** | ***** |
| p151-p160 | **-0.14** | **-0.19** | **-0.08** | ***** |
| DENSE (vs. SPARSE) |  |  |  |  |
| p131-p140 | **-0.05** | **-0.07** | **-0.02** | ***** |
| p141-p150 | **-0.06** | **-0.12** | **0.01** | **n.s.** |
| p151-p160 | **-0.07** | **-0.13** | **-0.02** | ***** |

Table A2: Results from the LMM analysis separately for the three subsets of participants.

|  | *Difference Contrast* | *Confidence Interval (Lower Bound)* | *Confidence Interval (Upper Bound)* | *Significance* |
| --- | --- | --- | --- | --- |
| FAST (vs. SLOW) |  |  |  |  |
| p131-p140 | **-12.7s** | **-16.8s** | **-8.9s** | ***** |
| p141-p150 | **-8.5s** | **-14s** | **-4.4s** | ***** |
| p151-p160 | **-12.6s** | **-16.4s** | **-9.2s** | ***** |
| DENSE (vs. SPARSE) |  |  |  |  |
| p131-p140 | **-5.5°** | **-8°** | **-3°** | ***** |
| p141-p150 | **-6.8°** | **-11.9°** | **-2.6°** | ***** |
| p151-p160 | **-8.7°** | **-14°** | **-3.5°** | ***** |

Figure A1:

Full distributions (smaller dots to the right), group-wide means (large dots to the left) and standard deviations (error bars) of fitted Kalman filter sensory noise parameters (i.e., the standard deviation of the normal distribution that represents the noise in perceiving the heading direction in any given moment), separately for each condition (x axis and color-coded) and the three participant subsets (A: p131-p140; B: p141-p150; C: p151-p160).


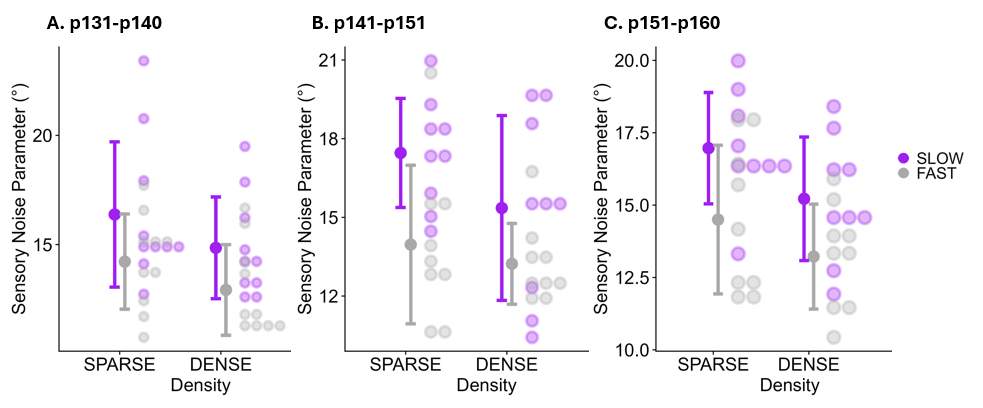


**Summary** – Both the LMM analysis (see Table A2) and the Kalman filter analysis (see Figure A1) showed consistent and robust results for all three sub-samples we tested here. The sub-sample effects were all in the same directions, and the 95% confidence intervals did (robustly) not include 0. The pictures is somewhat less consistent for the bootstrap analysis; particularly the effect of the density of optic flow on the Lag of Least Difference appeared to be vulnerable to a reduced sample size, with only one of the three sub-samples showing a significant result. However, the effect went in the same direction even in the two nonsignificant sub-samples.

Overall, statistical power appears to be extraordinarily high for the LMM analysis. However, in order to assess the effect of a manipulation on the participants’ responsiveness to changes in direction (i.e., the Lag of Least Difference), other types of analyses are needed, and at least our solution (the bootstrap analysis) appears to have a much lower statistical power. This is important to keep in mind for researchers who want to study the time course of their responses.

### B – Within-Participant Variability: Over the Course of a Run

In order to assess to what extent variability over the course of one run might affect the reliability of results, we divided each run into four parts of equal length and determined the Best Lag of Least Difference as well as the Least Difference for each of these four parts separately. We then correlated the fitted parameters for Part 1 with those of Part 2, Part 2 with Part 3, and Part 3 with Part 4. The results can be found in Figure A2 for the Lag of Least Difference and in Figure A3 for the Least Difference.

Figure A2:

Correlations between fitted Lags of Least Difference between first and second (A), second and third (B) and third and fourth (C) quarters of the participants’ runs, along with a fitted regression line. The inset shows the r squared for each correlation. D, E, F: As A, B, and C, but for the Normalized Least Differences.


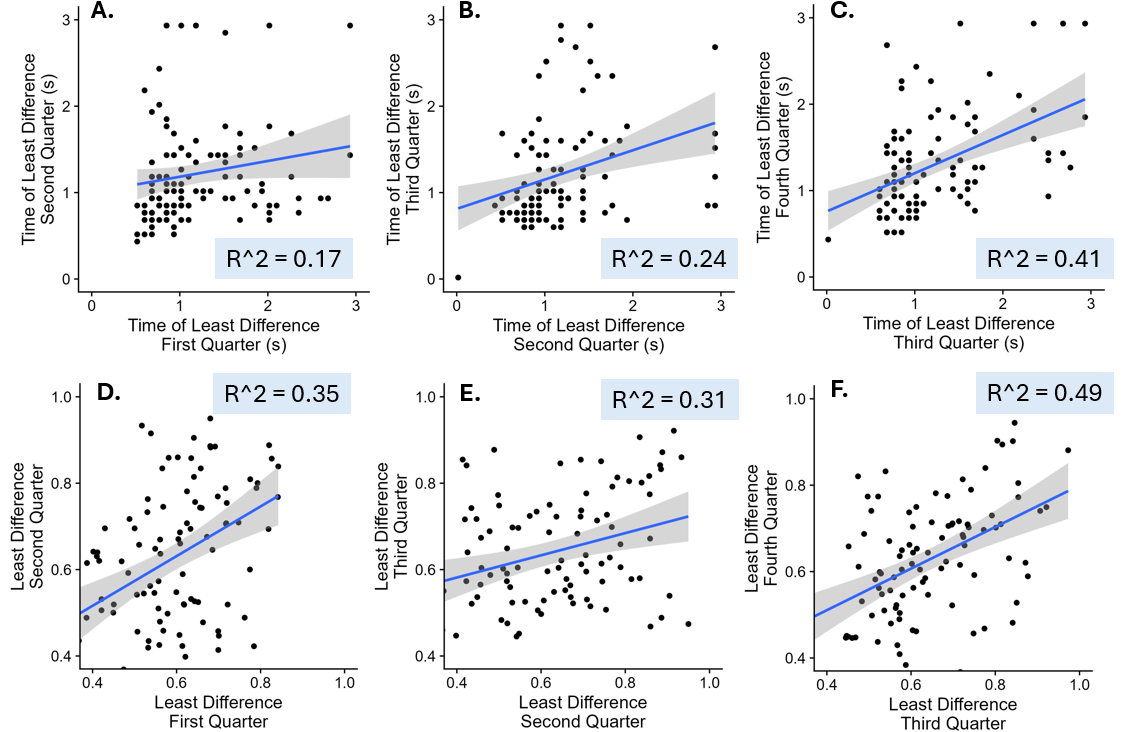


**Summary** – The correlations between the quarters generally increased for both measures (Lag of Least Difference and Normalized Least Difference) as the time in a run went on. This indicates that, over our short experiment, participant performance did not plateau or reach a final state; rather, their response continued to evolve. Further, the correlation even between the two last quarters was far from perfect; it was below 0.5 for both measures, which speaks to a considerable amount of within-participant variability.

Practically, these two observations show that it might be beneficial to increase the length of each run. This would allow participant performance to reach a stable state and further counteract within-participant variability with added statistical power.

### C – Within-Participant Variability: Robustness to Dropping Random Data Points

We finally also performed an individual-level bootstrap analysis where – very similar to the bootstrap analysis in the main body of this paper – we picked 1000 random samples of 25% of the data points from each participant and condition and determined the Lag of Least Difference and the Normalized Least Difference for each. We then took the 2.5^th^ and the 97.5^th^ percentiles for the fitted values as the lower and upper bound of (individual-level) 95% bootstrapped confidence intervals. Figure A3A and A3B show the individual-level estimates for the Lag of Least Difference and the Normalized Least Difference along with the 95% confidence intervals, while Figures A1C and A1D show the width of these 95% confidence intervals relative to the range of values across all conditions and participants as a measure of measurement variability.

Figure A3:

Results of the individual-level bootstrap analysis. A. Estimates of the Lag of Least Difference (dots) and 95% confidence intervals (error bars), separately for each participant (y axis) and condition (color-coded). B. As A but for the Normalized Least Differences. C. The distribution of the widths of the 95% confidence intervals across all participants, separately for each condition (color-coded). D. As C but for the Normalized Least Differences.


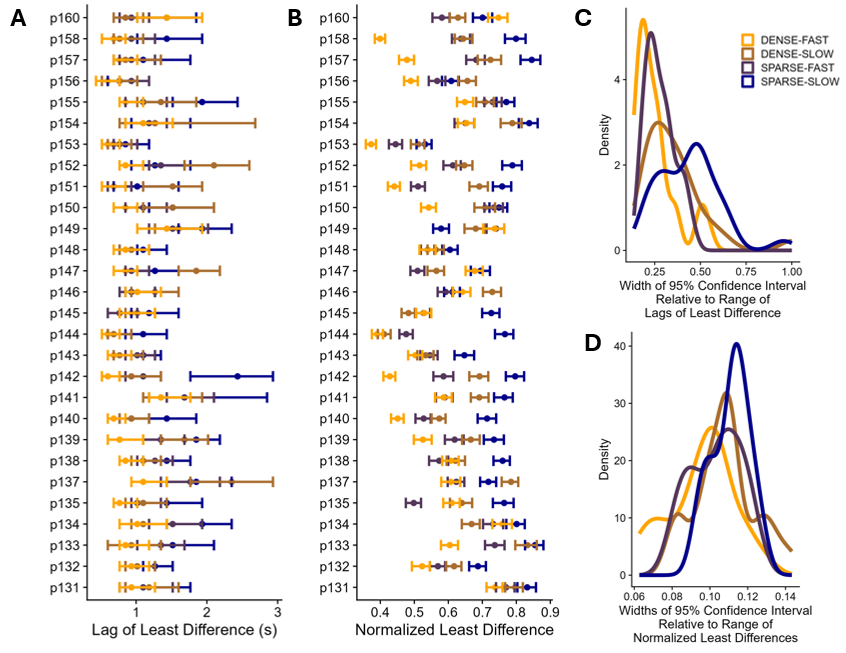


**Summary** – Overall, this procedure revealed that the Lag of Least Difference could, at least for some participants, change dramatically depending on which 25% of data points were included into the analysis (see, e.g., the SPARSE-SLOW condition for p142 in Figure A3A, or most conditions for p137). Figure A3C indicates that a large proportion of 95% individual-level confidence intervals covered 30% or more of the range of values. The picture was quite different for the Normalized Least Difference, which appeared to be fairly robust, with narrow 95% confidence intervals across all participants (see Figure A3B). Figure A3D shows that all 95% confidence intervals were narrower than 15% of the maximum range of observed values.

Overall, this analysis shows that data on variability in performance (as measured by the Normalized Least Difference) obtained through our method is highly reliable even for shorter runs. For the Lag of Least Difference (i.e., the measure of how long it took participants to react to changes in direction of self-motion) however, variability was much higher, which makes it more important to collect more data through, e.g., longer runs.
