# Supplementary material for: Precision and temporal dynamics in heading perception assessed by continuous psychophysics
Source: PLoS One. 2024 Oct 11;19(10):e0311992. doi: 10.1371/journal.pone.0311992 (PMC11469512; doi:10.1371/journal.pone.0311992)
Supplement: Attachment — Submitted filename: Response to Reviewers.docx [file pone.0311992.s002.docx]

**Reviewer #1**

Abstract:

Current models predict faster processing for faster and denser optic flow (OF). However, the focus has been on the accuracy of heading from OF rather than speed. The drift-diffusion model (DDM) presented in the introduction is not very representative of the research in heading from optic flow.

*1 – We did not intend to claim that DDMs play a central role in modelling space surrounding heading perception. Rather, we noticed some instances in which they were used without testing the full range of predictions derived from them. We made this clearer in the manuscript and make mention to other types of heading perception models.*

We validate a novel continuous psychophysics paradigm by replicating the effect of the speed and density of optic flow on precision. However, the precision found by the authors is well above previous results, and a direct comparison with more classical approaches is needed.

*2 – We have corrected the Kalman filter analysis and report the sensory uncertainty now – as is the norm – as standard deviation rather than variance.*

Main Manuscript:

L7-8: Sensory input (likelihood): It would be preferable to use the term "sensory evidence" and leave "likelihood" for the parameter being optimized in the Kalman (Kalman likelihood) to avoid confusion.

*3 – Amended.*

L10-12: This is odd. The only way to obtain a direct measure of observational uncertainty is to use the Kalman filter or linear quadratic Gaussian (LQG) models as in Straub et al. (2022). Unfamiliarity cannot be a justification for not using a Kalman-based approach.

*4 – We have clarified this section in the introduction, and we have also added a section in the discussion detailing the differences between the three types of analysis used in this paper, pointing out that only Kalman filters allow us to estimate observational uncertainty.*

L13-14: In this context, the authors should include citations from continuous psychophysics work (Knoll et al., and others) that test the tracking of the focus of expansion (FOE), which is a critical cue to recover heading from optical flow.

*5 – Thank for your drawing our attention to this line of research. We have added a discussion of two studies (Chow et al., 2021; Knöll et al., 2018) to the introduction.*

Stimulus Section: Here I have a major concern. The process noise has a standard deviation (SD) of 20 degrees, which is not justified. Such a large variability is well above the precision required to control heading by recovering the FOE in real-life situations.

*6 – One of the advantages of our method is that near-threshold stimulation is generally not required to obtain estimates of the differences in sensory noise (or overall performance in terms of precision and response lags). See for example (Bonnen et al., 2015) who found pronounced differences even between conditions where the dot participants had to track with their finger was clearly visible.*

Second, the change is made every 200 ms. Therefore, the structure is not a random walk, meaning that during 200 ms, observers can integrate flow consistent with a single direction. This is not a problem per se unless the Kalman model is adapted to these process dynamics. Unfortunately, there is no information in the paper concerning the implementation of the Kalman model.

*7 – We have conducted the Kalman filter analysis using 200 ms time windows (i.e., averaging participant performance over these time windows) rather than using a frame-by-frame updating mechanism. The results, which are reported in the Appendix to this review, are extremely similar to our original analysis (after correcting the mistake pointed out by reviewer #2). There may be arguments on a conceptual level, but given the negligible differences in results between both approaches, we believe it is preferable to maintain the frame-by-frame updating mechanism, which is methodologically more consistent with the bootstrapped CCG and LMM analyses.*

Data Analysis Section: - L19-20: The cross-correlogram (CCG) is run on angular positions (heading and response), which is not the usual way. The authors should have used the speed of changes to compute the CCG. However, the stimulus structure (changes every 200 ms) might interfere with the computation of the CCG, which assumes a new pair of values in each frame.

*8 – Indeed, if we were to use the speed of changes to compute the CCGs, we would have to average performance across time windows of 200 ms, which effectively leads to a huge drop in statistical power for the bootstrapped CCGs and the LMM analysis. We have performed this alternative analysis and, as we report in more detail in the Appendix to this response letter, we found effects in the same direction for these two analyses, without passing, however, the thresholds for statistical significance. The Kalman filter analysis was largely unaffected.*

*It is our opinion that the absence of statistically significant effects when using the speed of change (in comparison to angular position data) is due to the afore-mentioned drop in statistical power, not due to biases introduced by our original analysis. Arguably, an analysis based on angular position data is more prone to be influenced by differences in trajectories than an analysis based on the speed of change. However, given that we assigned the pre-generated trajectories randomly to each condition/participant, any such differences between the trajectories ultimately cancel out and not induce false positives. We have therefore maintained our original analysis but we have added a note that CCG analyses are generally based on the speed of change, as well the same explanation given here as to why we are of the opinion that our analysis is appropriate, and preferable in our case.*

Data Analysis Section - Kalman Filter: There is a total lack of information about the model, and I could not find the implementation in the OSF. The reported sensory noise is between 50 and 100 degrees, which are very large values. Given that the necessary information to evaluate the Kalman filter is missing, I am very unconfident that the analysis was conducted properly.

*9 – We have added more information on the Kalman filter in the manuscript. We have also split up the analysis into several scripts and named them more representatively, which should make it easier to find the relevant parts on OSF. Regarding the large estimates of sensory noise, we failed to account for the fact that our implementation of the model fitted the sensory noise parameter as variance rather than standard deviation. We have corrected this mistake and report the noise as standard deviation rather than as variance.*

**Reviewer #2**

1. To better understand the reasons for the deviation from the pre-registration, I have a question about the results from Experiment 1, which are described in the pre-registration document for experiment 2 (<https://osf.io/um7cg>). The document states that the authors "found no significant differences between any of the conditions for any of the experimental conditions for either of the two dependent variables". Table 1 in the document, however, indicates that the confidence interval of the difference contrasts between the fast and slow conditions exclude zero in for both the time lag and the normalized least error. Please clarify the statistical procedures used to assess significance.

*10 – Our apologies for this inconsistency in the pre-registration. We indeed used the confidence intervals to assess statistical significance there, and we did find that FAST led to lower time lags of least difference and normalized least errors in Experiment 1. To avoid further confusion, we have uploaded a corrected version of the pre-registration and will refer to this in the manuscript. We keep the original pre-registration on OSF and have added an explanation in the “wiki” section.*

2. The description of the Kalman filter analysis in the manuscript is very sparse. While it provides a good intuition about the model, some equations describing how the parameters were set or fit to the data would be helpful. After reading and executing the code, I have a couple minor concerns. Neither of these should invalidate the qualitative results in the present study, but they should be addressed in the interest of a proper use of the method and better interpretability of the values. a) Judging from the equations, which seem to follow the notation in Bonnen et al. (2015), the parameter Q should be the variance of the target random walk. In the manuscript (section "Stimulus"), the standard deviation of the target random walk is given as 20°, which would result in a variance of Q = 400°^2. Changing Q to this value in the code does not qualitatively change the effects, but it does change the concrete values for the sensory noise parameter. b) Following the previous point, the parameter R in the notation by Bonnen et al. (2015) should be the variance of the sensory noise in degrees^2, but the y axis of Figure 7 states the values as degrees. If we want to interpret the values as sensory uncertainty in the units of the experiment, reporting actual standard deviations is preferred. c) The procedure in the function KalmanFilter is based on random model simulations and is used to minimize the absolute error between the KF estimates and the subject's responses. The use of random simulations introduces variability in the obtained estimates due to the fitting and could be addressed by instead using the likelihood function in Appendix B of Bonnen et al. (2015) or in their code on <https://github.com/kbonnen/BonnenEtAl2015_KalmanFilterCode/blob/master/negLogLikelihoodr.m>.

*11 – We have added detail on the Kalman filter implementation in the manuscript and have fixed the mistakes and inconsistencies the reviewer has pointed out. (Thank you a lot!) We also implemented a maximum likelihood estimation to fit the parameters and report the sensory uncertainty as standard deviations.*

3. The CCG analysis seems non-standard. Typically, the cross-correlation between the velocity of the target and the velocity of the cursor is used, as in Mulligan (2002); Mulligan et al. (2013); Bonnen et al. (2015) and many other applications of tracking tasks. In the present manuscript, the authors compute the CCG on position instead of velocity, which might confound the dynamics of the target with the dynamics of the response. Furthermore, they use the mean absolute error between the target and response instead of the correlation. Is there a reason for these deviations from previous procedures? Since the target movement was the same across all conditions, I suspect that this will not systematically bias any of the conditions more than the others. However, to enable a comparison with other continuous psychophysics studies, I would like to additionally see the velocity CCGs and their statistics.

*12 – Since our stimulus only changes every 200 ms, using the change in heading angle over time means averaging participant responses over time windows of 200 ms, which severely impacts the power of our statistical analyses (particularly the bootstrapped CCGs and the LMM analysis). To our knowledge, using the heading angle (rather than the change in heading angle) might make differences in trajectories between the conditions more impactful. However, given that our pre-generated trajectories were assigned randomly across conditions and participants, such differences in the stimulus can’t account for the effects we found.*

*However, we certainly acknowledge that the standard procedure is to use the change in heading and have added a section in the manuscript laying out the reasons for this choice. We further, as requested, report the results of the analysis based on the change in heading in the appendix to this review; in a nutshell, the pattern in results remains comparable in direction but nor the bootstrapped CCGs nor the LMM analysis pass the significance threshold.*

As mentioned earlier, none of these methodological concerns are likely to affect the qualitative effects on which the conclusions are based. This is an interesting application of continuous psychophysics to a naturalistic task, which will be a valuable addition to the literature once the minor points above are addressed.

*13 – Thank you!*

As a final point, I want to comment on the comparison between the Kalman filter and the CCG as different analysis approaches for continuous psychophysics. The introduction states that "reliance on Kalman filter modelling may hinder adoption of continuous psychophysics, either because researchers may not be familiar with this approach or because they prefer not to buy into the assumptions underlying a Kalman filter". This is not a good reason against the use of a method, because there is no model without assumptions. On the contrary, I would view it as an advantage of Kalman filters and other probabilistic approaches that they transparently state their assumptions. On the other hand, it is less clear which assumptions underlie the interpretation of the peak or the lag of a CCG as a measure of perceptual precision or processing speed. Furthermore, multiple studies have emphasized the need to model the motor system in addition to a perceptual model like the Kalman filter (Ambrosi et al., 2022; Straub et al., 2022; Falconbridge et al., 2023), since these factors also affect behavior. If the goal is to obtain quantitative estimates from continuous psychophysics that can be clearly attributed to perceptual, cognitive, and motor processes (like sensitivity and criterion in SDT), these factors need to be taken into account. Besides these theoretical considerations, there are also empirical differences between the measuremements obtained from the different analysis methods. In the discussion, the authors say: "Continuous Psychophysics is an excellent tool to study the precision of participant responses, be it using a Kalman filter-based approach or the LMM analysis suggested in this paper." While the qualitative effects in the present study are the same between the KF and the CCG summary statistics, it looks like they do capture slightly different aspects of participants' behavior. If the authors would like to provide recommendations on the preferred analysis methods for future continuous psychophysics studies, a more detailed examination of the differences between the results obtained from the different analysis approaches and their robustness, would be appropriate.

*14 – We agree with reviewer’s observations. It is important to be precise (and conservative) when drawing conclusions from (perhaps superficial) methods like the CCG and LMM-based analyses, while the theoretical commitments of the Kalman filter analysis are likely worth it under many circumstances. We have added a section in the discussion in which we address these notions.*

*We now also address the absence of a motor component when modelling our results (as suggested by the studies pointed out by the reviewer) as a limitation of the present study.*

References:
- Bonnen, K., Burge, J., Yates, J., Pillow, J., & Cormack, L. K. (2015). Continuous psychophysics: Target-tracking to measure visual sensitivity**.** *Journal of vision*, *15*(3), 14-14.
- Mulligan, J. B. (2002). Sensory processing delays measured with the eye-movement correlogram. *ANNALS-NEW YORK ACADEMY OF SCIENCES*, *956*, 476-478.
- Mulligan, J. B., Stevenson, S. B., & Cormack, L. K. (2013, March). Reflexive and voluntary control of smooth eye movements. In *Human Vision and Electronic Imaging XVIII* (Vol. 8651, pp. 223-244). SPIE.
- Ambrosi, P., Burr, D. C., & Cicchini, G. M. (2022). Ideal observer analysis for continuous tracking experiments. *Journal of Vision*, *22*(2), 3-3.
- Straub, D., & Rothkopf, C. A. (2022). Putting perception into action with inverse optimal control for continuous psychophysics. *Elife*, *11*, e76635.
- Falconbridge, M., Stamps, R. L., Edwards, M., & Badcock, D. R. (2023). Continuous psychophysics for two-variable experiments; A new “Bayesian participant” approach. *i-Perception*, *14*(6), 20416695231214440.

***Appendix***

In the following, we briefly outline the results of our bootstrapped CCG and LMM analyses when using the change in heading angle over time rather than the heading angle itself. Please note that our stimulus, which only changes every 200ms (or 12 frames), forces us to average participant performance over these time windows (see Figure 1). This lowers statistical power drastically and prevents us from obtaining smooth CCGs (see Figure 2 in this Appendix in comparison to Figure 5 in the manuscript).

Figure 1: Presented heading (black) and mean participant response for each 200ms time interval (red) for two participants (rows of panels) in the same condition (SLOW-DENSE). This corresponds to Figure 4 in the manuscript.

Figure 2: CCGs for two participants (rows of panels) and all conditions (color-coded and columns of panels). This corresponds to Figure 5 in the manuscript.

Figure 3: Full distributions of Time Lags of Max Correlation (A) and Max Correlations (B). This corresponds to Figure 6 in the manuscript.

Table 1: Difference contrasts and corresponding confidence intervals for the bootstrapped CCG analysis. This corresponds to Table 1 in the manuscript.

|  | *Difference Contrast* | *Confidence Interval (Lower Bound)* | *Confidence Interval (Upper Bound)* | *Significance* |
| --- | --- | --- | --- | --- |
|  | ***Time Lag of Least Difference***  *(Intercept = 1.54)* | | | |
| FAST (vs. SLOW) | **-0.22s** | **-0.52s** | **0.22s** | **n.s.** |
| DENSE (vs. SPARSE) | **-0.21s** | **-0.44s** | **0.31s** | **n.s.** |
|  | ***Max Correlation***  *(Intercept = 0.11)* | | | |
| FAST (vs. SLOW) | **0.012** | **-0.026** | **0.06** | **n.s.** |
| DENSE (vs. SPARSE) | **0.01** | **-0.026** | **0.066** | **n.s.** |

**Results: Bootstrapped CCGs –** None of the difference contrasts are statistically significant, but they go in the expected direction. Please note that the correlation is expected to higher (unlike the normalized least error in the manuscript) for the FAST (than for SLOW) and DENSE (than for SPARSE) conditions.

**Results: Linear Mixed Modelling** – The intercept for the LMM was 99.6 °/s (95% CI = [97.4 °/s; 102 °/s]). The absolute difference between the presented and the response change in heading angle per second was (non-significantly) lower by 2.4 °/s (95% CI = [-5.7 °/s; 1.1 °/s]) for FAST than for SLOW, and (non-significantly) lower by 1.3 °/s (95% CI = [-5.1 °/s; 2.5 °/s]) for DENSE than for SPARSE.

Figure 4. Results of the Kalman filter when simulating updating in 200 ms windows. This corresponds to Figure 7 in the manuscript.

**Results: Kalman Filter –** The results when averaging across 200 ms time windows are extremely similar to the original analysis.

The code used for these analyses can be found in the (updated) data and code package (<https://osf.io/vb9xs>) in the script “Revs - Analysis Change in Angle.R”.
