# Supplementary material for: Precision and temporal dynamics in heading perception assessed by continuous psychophysics
Source: PLoS One. 2024 Oct 11;19(10):e0311992. doi: 10.1371/journal.pone.0311992 (PMC11469512; doi:10.1371/journal.pone.0311992)
Supplement: Attachment — Submitted filename: Response to Reviewers 2.docx [file pone.0311992.s003.docx]

**Response to the Reviewers**

Reviewer #1: I appreciate the efforts made by the authors to address my previous concerns. However, there are two issues (one more critical) that still need to be addressed:

a) Model Fit Methodology: Upon reviewing the analysis code, it appears that the fit of the Kalman filter is not based on minimizing the negative log-likelihood as the authors claim in the manuscript, as would be expected in order to obtain an optimal estimate. Instead, the authors have opted to minimize the mean error between the response and the Kalman posterior estimate. This method does not use the square of the error or even the root mean square error (RMSE), which raises concerns about the validity of the estimates. This approach does not typically guarantee that the estimated parameters are the most likely given the data, which could potentially lead to unreliable conclusions. This relates to point b.

*RESPONSE #1: We followed the procedure outlined by Bonnen et al. (2015), which implements a negative log likelihood estimation. We are assuming that the modelling fitting step the reviewer refers to is the one where we first determine the errors between predicted and observed values (line 64 in Analysis Kalman filter.R). We then determine the negative log likelihood that these values come from a normal distribution with a mean of 0 and a standard deviation of K^2 * R, as Bonnen et al describe it in lines 19 – 22 of their matlab script (*[*https://github.com/kbonnen/BonnenEtAl2015_KalmanFilterCode/blob/master/negLogLikelihoodr.m*](https://github.com/kbonnen/BonnenEtAl2015_KalmanFilterCode/blob/master/negLogLikelihoodr.m)*).*

b) Estimate Sizes: The parameter estimates provided are unusually large (about 15degs), which seems implausible in the context of the studied phenomena. This issue might suggest a scaling error in the data or model mis-specification. It is crucial that the authors reassess these estimates to ensure they are realistic and reflect the true dynamics of the data. One possibility is that motor noise is being affecting the estimates, but I doubt it. The authors should test how the process noise value is affecting their estimates, but through a true minimization of the neg likelog, which is absent in the paper.

I recommend that the authors re-evaluate these aspects of their methodology to ensure the robustness and validity of their findings.

*RESPONSE #2: We have double-checked our code and corrected a concrete mistake spotted by reviewer #2. This has, however, not lowered the parameter estimates significantly. We are confident in this version of the code, and given how hard the task was for some participants (anecdotally), we believe that these values are sensible. Furthermore, what matters for our conclusions are the differences between the conditions, which are clear, and in line with the results from the CCGs. (See, e.g., Bonnen 2015, who found similarly striking differences in best time lag and peak correlation between their conditions, which were correlated strongly with the fitted Kalman filter sensory noise estimates.) Further, heading discrimination is much less sensitive outside of a +-45° window around the straight-ahead* (Gu et al., 2010)*; only 25% of our frames fell into this more sensitive window. Consequently, since our analysis implicitly averages across all heading angles, the overall sensitivity estimates should therefore be much higher (indicating lower precision) than the 1-2° reported by Gu et al. (2010) for the straight-ahead. We have, nonetheless, noted the reviewers concern in the discussion as readers should be aware of this mismatch between the literature and our study, along with some tentative explanations.*

Reviewer #2: The revised version addresses most concerns I had about the original manuscript but makes a few of its problems more apparent. In particular, I have two minor points about the description of the KF analysis and the stimulus dynamics. The final point concerns the statements about the assumptions of different models and why I think the authors do not do themselves a favor by making these statements or bringing up the DDM. For each point, I make recommendations for how to address it.

1.The Kalman filter implementation has been fixed and the code now seems to implement a maximum likelihood estimate. The authors have added a bit of description of the model in the paper. Here, I am confused by one particular sentence: "The participant response at t+1 is then modelled as presented heading at t multiplied by the Kalman gain K". This is inconsistent with the version of the KF model of continuous psychophysics by Bonnen et al. (2015), where the subject's response is assumed to be the KF estimate $\hat x$. This could be clarified by writing out the equations for the KF, i.e. how the belief is updated, and specifying exactly which variables are assumed to correspond to behavior and which to the state of the experiment.

*RESPONSE #3: We have corrected this mistake in the code and now properly conduct the Kalman filter fitting in line with Bonnen et al. (2015). We have also corrected the phrasing in the manuscript and have added some further detail in the modelling section.*

2.In response to comments by both reviewer 1 and me about the non-standard CCG analysis, the authors clarified that a standard velocity CCG analysis was not possible because of the target dynamics in their task, which operates at a time step of 200ms, while the behavioral response has a higher frame rate. This is a limitation of the experimental design of the present study and should be discussed as such.

*RESPONSE #4: We have added this is in the discussion section.*

3.Both reviewer 1 and I criticized the statement in the original version of the manuscript that "reliance on Kalman filter modelling may hinder adoption of continuous psychophysics, either because researchers may not be familiar with this approach or because they prefer not to buy into the assumptions underlying a Kalman filter." The authors have now slightly rearranged that paragraph. They have removed unfamiliarity of researchers with the approach as a reason for not adopting the modeling approach, which is commendable. Still, the revised version states "the cost of buying into the assumptions underlying this category of model" as a reason for not using models based on Bayesian inference and optimal control and that "other analysis methods require fewer theoretical commitments".
I want to push back on this because, in the revised version, both the introduction and discussion feature the drift diffusion model (DDM) as a potential model for modeling perception in continuous tasks. The revised introduction even states that "this study focusses on one particular class of models that has been applied to heading perception: Drift Diffusion Models". This is confusing, because there is no analysis based on DDMs anywhere to be found in the paper. More importantly, though, the DDM also makes assumptions, like any model does. There is typically an algorithmic process of noisy evidence accumulation towards a bound with particular assumptions about the functional form of noises, drift rates, and bounds. If the authors want to criticize one class of models for making assumptions while favoring another class of models, they could start a discussion about why one set of assumptions is preferable over another based on theoretical or empirical considerations. But simply stating that adoption of one class of models is hindered because people "prefer not to buy into the assumptions" is not going to suffice, especially when the present manuscript provides no argument (theoretical or empirical) for why DDMs might be more appropriate for continuous psychophysics tasks. Both the comment about the assumptions of KF and LQG as models of CPP and the statements about the DDM contribute nothing of value to the paper and should be removed, in my opinion. If the authors insist on making comments about the assumptions of different models, I would expect a better justification. Similarly, if the DDM really was a central focus of the paper, I would expect to see it in effect for the task at hand.

*RESPONSE #5: We agree that DDMs occupied an outsized space in the introduction and have cut down our mention of them to the minimum required to convey (one of the) motivations for this study: DDMs predict faster judgements for more informative optic flow, a prediction that has not been tested yet.*

*Further, we have clearly failed to properly express our opinions regarding the assumptions underlying the Kalman filter in the paper. Our intention was never to claim that DDMs make fewer (or even no) assumptions in comparison to a Kalman filter. Rather, we propose the (bootstrapped) CCG-based analysis and the LMM analysis as alternatives that requires fewer assumptions (namely they can be used without assuming a Bayesian updating process) but also allow for less specific conclusions (we can only report the maximum correlations and best time lags obtained via CCGs, or changes in precision via the LMM, but we can NOT draw any conclusions about the uncertainty in the participant’s representation of the stimulus). We have amended these sections to make our comparison clearer.*

**References**

Bonnen, K., Burge, J., Yates, J., Pillow, J., & Cormack, L. K. (2015). Continuous psychophysics: Target-tracking to measure visual sensitivity. *Journal of Vision*, *15*(3). https://doi.org/10.1167/15.3.14

Gu, Y., Fetsch, C. R., Adeyemo, B., DeAngelis, G. C., & Angelaki, D. E. (2010). Decoding of MSTd Population Activity Accounts for Variations in the Precision of Heading Perception. *Neuron*, *66*(4), 596–609. https://doi.org/10.1016/j.neuron.2010.04.026
