# Supplementary material for: Precision and temporal dynamics in heading perception assessed by continuous psychophysics
Source: PLoS One. 2024 Oct 11;19(10):e0311992. doi: 10.1371/journal.pone.0311992 (PMC11469512; doi:10.1371/journal.pone.0311992)
Supplement: Attachment — Submitted filename: Response to Reviewer.docx [file pone.0311992.s004.docx]

Reviewer #1: The manuscript has improved clarity in some aspects, but a couple of key issues remain.

First, the type of negative log-likelihood (negloglik) that the authors are minimizing is not based on maximizing the Kalman log-likelihood, but rather on the negloglik of the residuals (i.e., the difference between the response and the posterior). While this is now clear in the text and not technically incorrect, the authors continue to cite Bonnen et al. (2015) as if they are using the same procedure, which is misleading. In Bonnen’s paper, they maximize the log-likelihood of the Kalman itself (equations B15/B16) by fitting the observational noise parameter R , which can be interpreted as visual precision. This is fundamentally different from what the current manuscript is doing. While I still believe this could affect the estimates, I will not pursue this point further. However, the authors should correct this misrepresentation and clearly distinguish between the two approaches, ensuring that Bonnen et al. is cited appropriately.

1. *Bonnen et al. (2015) use the approach that we have implemented, as described/justified in a comment in their code* [*https://github.com/kbonnen/BonnenEtAl2015_KalmanFilterCode/blob/master/negLogLikelihoodr.m*](https://github.com/kbonnen/BonnenEtAl2015_KalmanFilterCode/blob/master/negLogLikelihoodr.m)*:*

*We have now made this explicit in our manuscript.*

Second, the manuscript misleadingly presents LMM as a novel method for measuring precision, which is not accurate. The essence of continuous psychophysics (CPP) is to derive a measure of precision, often equivalent to Just Noticeable Differences (JND) or differential thresholds. The manuscript uses the term “precision” loosely in several sections. In the context of sensory processing, precision refers to the inverse of variance or uncertainty, as typically modeled by Kalman Filters and Bayesian approaches (or JND in classical psychophysics). The authors should avoid conflating this concept with what LMM captures. While LMM is appropriate for analyzing condition-based differences in performance, it does not estimate sensory noise or uncertainty as Kalman Filters do. LMMs, as used in the manuscript, are not designed to estimate precision in the context of sensory processing. Instead, they only reveal significant differences between conditions based on absolute error—accuracy would likely be a more appropriate metric here. The claim that LMM offers a complementary method to the Kalman Filter for measuring precision is incorrect and needs to be revised. Clarifying these methodological distinctions would enhance the manuscript’s clarity and main contribution.

I suggest that the authors focus on LMM as a tool for detecting performance differences between conditions based on error or accuracy. The manuscript’s primary focus should shift toward this type of analysis, reframing LMM’s role in understanding performance variation, without suggesting that it measures precision. While this approach may be less exciting than measuring variability or sensory precision, it is a more accurate representation of what is the main contribution of the study.

2. *We agree that it is extremely important to keep apart sensory uncertainty as measured by, e.g., the Kalman Filter, and performance differences as measured by alternative methods of analyzing such as our LMM approach. We are keenly aware of this difference and have now made this clearer in the manuscript as follows:*

*We have changed “precision” to “variability in performance” throughout unless we are talking specifically about sensory uncertainty (as determined by the Kalman filter). We don’t think “accuracy” would communicate our intentions appropriately because, in our understanding, the terms refers to (systematic) biases rather than (random) variability or noise in performance. While there are applications for an investigation into biases, this project was set up to assess variability in performance in the different conditions.*

Finally, the discussion should also be revised to reflect these clarifications.

3. *We have made the appropriate changes in the discussion as well.*
